# Local Recurrence After Sublobar Resection for Primary Lung Cancer: Does the Type of Stapling Device Matter?

**DOI:** 10.1093/icvts/ivaf171

**Published:** 2025-07-25

**Authors:** Go Kamimura, Masaya Aoki, Satomi Imamura, Shoichiro Morizono, Takuya Tokunaga, Tadashi Umehara, Aya Harada-Takeda, Koki Maeda, Toshiyuki Nagata, Kazuhiro Ueda

**Affiliations:** Department of General Thoracic Surgery, Kagoshima University Graduate School of Medical and Dental Sciences, Kagoshima 890-8520, Japan; Department of General Thoracic Surgery, Kagoshima University Graduate School of Medical and Dental Sciences, Kagoshima 890-8520, Japan; Department of General Thoracic Surgery, Kagoshima University Graduate School of Medical and Dental Sciences, Kagoshima 890-8520, Japan; Department of General Thoracic Surgery, Kagoshima University Graduate School of Medical and Dental Sciences, Kagoshima 890-8520, Japan; Department of General Thoracic Surgery, Kagoshima University Graduate School of Medical and Dental Sciences, Kagoshima 890-8520, Japan; Department of General Thoracic Surgery, Kagoshima University Graduate School of Medical and Dental Sciences, Kagoshima 890-8520, Japan; Department of General Thoracic Surgery, Kagoshima University Graduate School of Medical and Dental Sciences, Kagoshima 890-8520, Japan; Department of General Thoracic Surgery, Kagoshima University Graduate School of Medical and Dental Sciences, Kagoshima 890-8520, Japan; Department of General Thoracic Surgery, Kagoshima University Graduate School of Medical and Dental Sciences, Kagoshima 890-8520, Japan; Department of General Thoracic Surgery, Kagoshima University Graduate School of Medical and Dental Sciences, Kagoshima 890-8520, Japan

**Keywords:** local recurrence, automatic stapling device, nondisposable knife type, disposable knife type

## Abstract

**Objectives:**

Two major types of stapling devices exist: those with disposable built-in knives and those with nondisposable built-in knives. This study investigated whether the stapler type influences the incidence of local recurrence, including margin recurrence and pleural dissemination, after curative sublobar resection for lung cancer.

**Methods:**

We retrospectively reviewed patients who underwent sublobar resection at our institution between 2010 and 2021. We compared disease-free survival, overall survival, and local recurrence between procedures using a stapler with a disposable knife and those using a stapler with a nondisposable knife.

**Results:**

A total of 269 patients were included: 84 were treated with the disposable-knife stapler and 185 with the nondisposable-knife stapler. Local recurrence occurred in 22 of 269 patients (8.2%), including 9 of 84 (10.7%) in the disposable group and 13 of 185 (7.0%) in the nondisposable group (*P* = .72). Patients who developed local recurrence tended to be older, male, have a smoking history, squamous cell carcinoma, absence of a ground-glass component, positive stapling cartridge cytology, partial resection, right lower lobe tumours, elevated carcinoembryonic antigen, and higher maximum standardized uptake values. In a propensity score-matched study (78 patients per group), no significant differences in disease-free survival, overall survival, or local recurrence were detected between the stapler types.

**Conclusions:**

No statistically significant differences in oncological outcomes were observed between stapler types in this retrospective study; however, the absence of a significant difference does not rule out a real effect. Further large-scale research is warranted.

## Graphical Abstract

**Figure:**
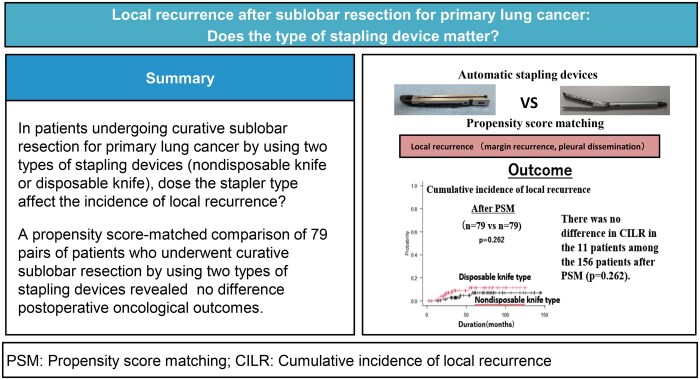

## INTRODUCTION

**Figure 1.**
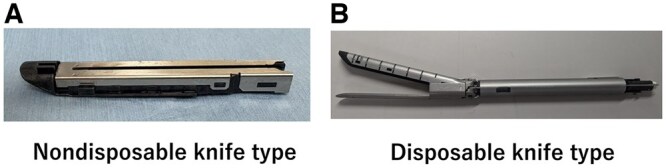
Two Types of Automatic Stapling Device Are Used in the Field of Thoracic Surgery. In nondisposable knife type devices (A) only the staples are replaced, while in disposable knife type devices (B) both the staples and the knife are replaced. The photographs shown were taken by the authors.

Automatic stapling devices that are frequently used in the general thoracic surgery field can be divided into 2 types depending on their structure: the nondisposable knife type, in which only the staple is replaced (**Figure 1A**), and the disposable knife type, in which the staple and knife are replaced (**Figure 1B**).

Local recurrence that occurs in association with the use of automatic stapling devices consists of stump and pleural dissemination recurrence.^1^^,^^2^ Previous reports of local recurrence in sublobar resection have mentioned the length of the surgical margin^3–6^ and the cytology of stump staples^7–10^; however, there have been no reports focusing on the different types of automatic stapling devices.

In the field of gastrointestinal surgery, there have been several reports of recurrence due to implantation of automatic stapling devices.^11–13^ When using a nondisposable knife type staple device, if cancer cells adhere to the knife, there is a risk that cancer cells will be implanted the next time it is used.^14^^,^^15^

Therefore, we focused on curative sublobar resection for lung cancer^16^^,^^17^ with a risk of local recurrence and investigated whether the type of the automatic stapling device influenced the incidence of local recurrence.

## METHODS

### Study design and participants

Among 1606 patients who were diagnosed with non-small cell lung cancer (NSCLC) and then surgically treated at our hospital between January 2010 and December 2021, 269 patients with NSCLC who underwent curative sublobar resection were included. To secure an adequate sample size for meaningful statistical analysis, this study included patients treated at our institution between 2010 and 2021. During this entire period, both disposable and nondisposable stapling devices were consistently available. Furthermore, there were no design changes to either device, and their relative usage frequency remained stable over time. This continuity allowed us to compare outcomes between the 2 stapler groups without introducing bias from evolving practices or learning-curve effects.

Since 2010, the majority of surgeries in this study were performed using video-assisted thoracoscopic surgery, with open thoracotomy reserved for select cases based on tumour location and patient condition. For intersegmental plane identification, the inflation-deflation method was primarily used. Additionally, since 2015, near-infrared imaging with indocyanine green (ICG) has been actively utilized for segmentectomy to achieve more precise delineation. These surgical approaches remained consistent throughout the study period, and the core surgical team also remained largely unchanged, minimizing variability related to surgeon experience or personnel shifts.

This retrospective clinical study was approved by the Kagoshima University Hospital Ethics Committee (approval number: 230234epi). This was a retrospective study and no interventions were performed for research purposes. Then the research participants and their relatives could opt out by viewing the research content hosted online. The study complied with the principles of the Declaration of Helsinki. Pathological tumour-node-metastasis staging was recorded for all patients based on the 8th edition of the American Joint Committee on Cancer/Union for International Cancer Control classification.^18^

**Figure 2.**
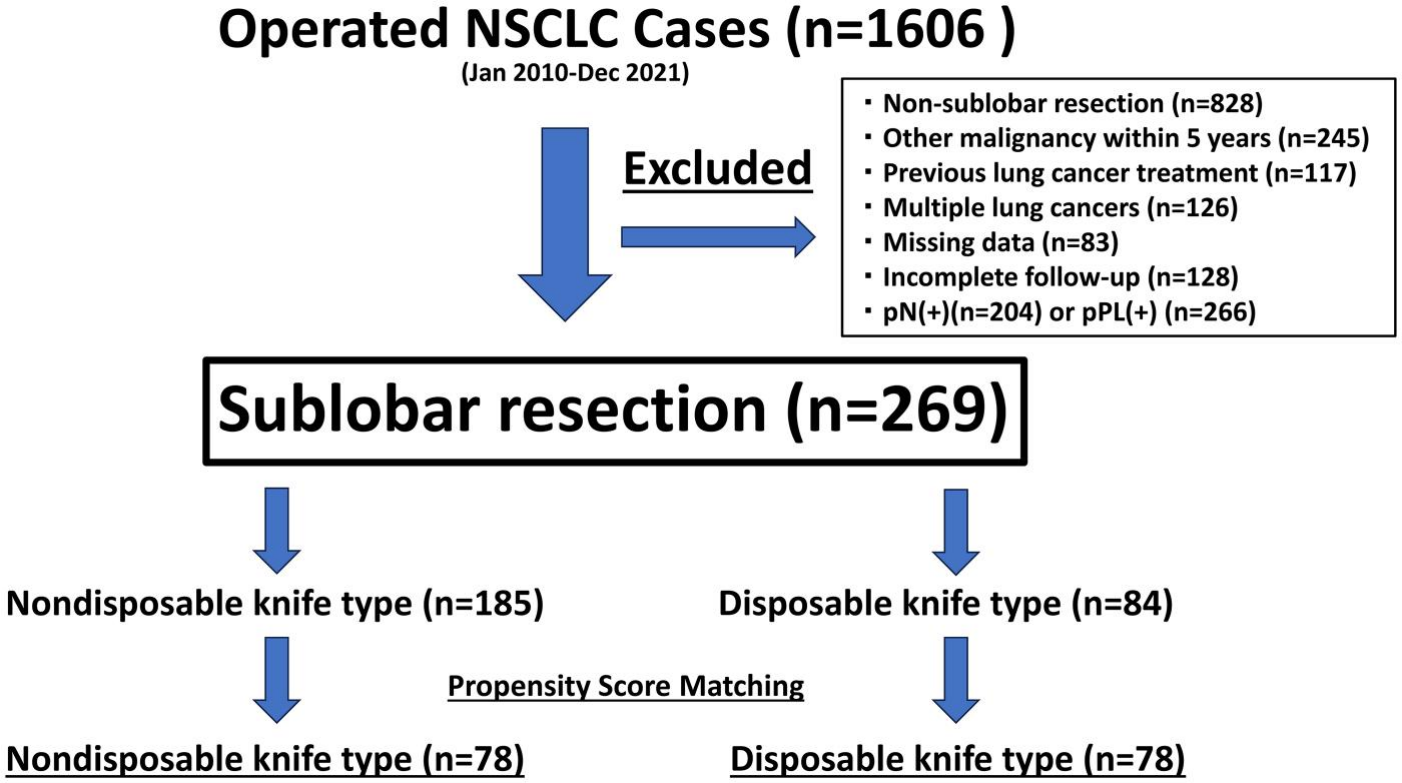
Flowchart of Patient Selection for the Present Study.

Sublobar resection was performed for lung cancer with clinically invasive size of ≤30 mm for which a surgical margin of ≥10 mm could be secured. Segmentectomy of multiple segments was also permitted to ensure the surgical margins. When using a nondisposable knife-type automatic stapling device, the cartridge was washed with distilled water before it was replaced. The type of stapler used was primarily determined by surgeon preference, availability, and the institution’s standard practice at the time of surgery. The exclusion criteria were as follows: (a) surgical method other than sublobar resection (*n* = 828); (b) history of treatment for other malignant tumours within 5 years (*n* = 245); (c) history of lung cancer treatment (*n* = 117); (d) multiple lung cancers (*n* = 126); (e) data not measured (*n* = 83) (in this study, “data not measured” indicates that key variables were entirely absent from the original records, rendering imputation methods unworkable); and (f) lack of complete follow-up information (*n* = 128). In this study, in order to accurately examine local recurrence caused by automatic stapling devices, patients with pN (+) (*n* = 204) and pPL (+) (*n* = 266) disease were excluded. The patient selection flowchart for this study is presented in **Figure 2**.

### Statistical analyses

Comparisons between the 2 groups were conducted using standardized mean difference (SMD). The median follow-up period was analysed using the reverse Kaplan-Meier method. Patient survival curves were plotted using the Kaplan-Meier method, and the difference between survival curves was analysed using a log-rank test. Overall survival was defined as the time from the date of surgery to the date of death (any cause). Disease-free survival (DFS) represented the duration between the surgical date and the date of recurrence or death. The risk of local recurrence was expressed as a linearized risk (events per 100 person-years) to accurately account for differences in follow-up duration between groups. The cumulative incidence of local recurrence (CILR) was examined using a Gray competing risk analysis. The cumulative incidence of local recurrence was defined as the interval from the date of surgery to the date of local recurrence of the disease, censored for patients without events at their last clinic visit. Local recurrence events were analysed using linearized risk and Gray’s competing-risk analysis, treating death as a competing event. Disease-free survival, which incorporates all recurrence events (local or distant) and death, was analysed separately with Kaplan-Meier curves and log-rank tests. In the propensity-score-matched cohort, CILR was compared between stapler types using a pair-stratified Gray’s test.

Propensity scores were calculated by logistic regression using 14 baseline characteristics (age, sex, smoking status, histological type, clinical tumour size, clinical invasive size, ground-glass opacity, histological grade, surgical procedure, tumour location, serum carcinoembryonic antigen, maximum standardized uptake value on positron emission tomography, Ly, and V). Patients were matched in a 1:1 ratio using the nearest neighbour method with the calliper value set at 0.2. All statistical analyses were performed using EZR (Saitama Medical Center, Jichi Medical University, Saitama, Japan) and SPSS (SPSS II for Windows, Standard Version 26.0; SPSS Inc., Chicago, IL, USA). *P* values of <.05 were considered to indicate statistical significance.

## RESULTS

**Table 1** shows the characteristics of all 269 patients before propensity score matching (PSM). Among the measured variables, the greatest imbalance was observed in the number of staples used to cut the lung (SMD = 0.280). The nondisposable knife group also showed a slightly larger pathological tumour size (SMD = 0.159), whereas the local recurrence rate (SMD = 0.130) and the proportion of patients undergoing additional resection (SMD = 0.036) indicated smaller differences between the 2 groups.

**Table 1.** Clinical and Pathological Findings of All Patients Before Propensity Score Matching

			Automatic stapling device		
Factors		Total (n = 269) (%)	Nondisposable (n = 185) (%)	Disposable (n = 84) (%)	SMD
Age	Median ± SD	71 ± 8.9	70.5 ± 9.1	72.0 ± 8.6	0.104
Sex	Male	124 (46.1)	81 (43.8)	43 (51.2)	−0.149
	Female	145 (53.9)	104 (56.2)	41 (48.8)	0.149
Smoking	Never	139 (51.7)	97 (52.4)	42 (50.0)	0.049
	Ever	130 (48.3)	88 (47.6)	42 (50.0)	−0.049
Histological type	AD	223 (82.5)	155 (83.8)	68 (80.9)	0.074
	SCC	32 (11.9)	20 (10.8)	12 (14.3)	−0.105
	Others	14 (5.6)	10 (5.4)	4 (4.8)	0.030
Clinical tumour size	Median ± SD (mm)	16.0 ± 6.6	15.0 ± 6.9	17.0 ± 6.3	−0.130
Clinical invasive size	Median ± SD (mm)	7.0 ± 8.2	6.6 ± 8.2	7.0 ± 8.0	−0.024
GGO	(+)	181 (67.3)	124 (67.0)	57 (67.9)	−0.018
	(−)	88(32.7)	61 (33.0)	27 (32.1)	0.018
Pathological tumour size	Median ± SD (mm)	15.0 ± 7.2	15.0 ± 7.5	15.0 ± 6.4	0.159
Vascular invasion	(+)	14 (5.2)	8 (4.3)	6 (7.1)	−0.121
	(−)	255 (94.8)	177 (95.7)	78 (92.9)	0.121
Lymphatic invasion	(+)	19 (7.1)	12 (6.5)	7 (8.3)	−0.070
	(−)	250 (92.9)	173 (93.5)	77 (91.7)	0.070
Histological grade	G1	166 (61.7)	117 (63.3)	49 (58.4)	0.100
	G2	77 (28.6)	50 (27.0)	27 (32.1)	−0.110
	G3	19 (7.1)	12 (6.5)	7 (8.3)	−0.070
	G4	7 (2.6)	6 (3.2)	1 (1.2)	0.140
Stapling cartridge lavage cytology	(+)	12 (4.5)	8 (4.3)	4 (4.8)	−0.021
	(−)	163(60.6)	113 (61.1)	50 (59.5)	0.032
	Unknown	94 (34.9)	64 (34.6)	30 (35.7)	−0.023
Surgical procedure	Wedge	153 (56.9)	112 (60.5)	41 (48.8)	0.237
	Segmentectomy	116 (43.1)	73 (39.5)	43 (51.2)	−0.237
Number of staples used	Median ± SD	4.0 ± 1.1	4.0 ± 1.2	3.0 ± 1.0	−0.280
Additional resection	(+)	4 (1.5)	3 (1.6)	1 (1.2)	0.036
	(−)	265 (98.5)	182 (98.4)	83 (98.8)	−0.036
Tumour location	RU	63 (23.4)	48 (25.9)	15 (17.9)	0.196
	RM	8 (3.0)	6 (3.2)	2 (2.4)	0.052
	RL	67 (24.9)	40 (21.6)	27 (32.1)	−0.239
	LU	82 (30.5)	58 (31.5)	24 (28.6)	0.061
	LL	49 (18.2)	33 (17.8)	16 (19.0)	−0.031
CEA value	Median ± SD	2.6 ± 2.9	2.6 ± 2.9	2.8 ± 2.9	0.094
SUV max value	Median ± SD	1.4 ± 2.8	1.4 ± 2.8	1.3 ± 2.7	0.096

Abbreviations: Median±standard deviation, Median[minimum-maximum]; SMD, standardized mean difference; AD, adenocarcinoma; CEA, carcinoembryonic antigen; GGO, ground-glass opacity; LL, left lower lobe; LU, left upper lobe; RL, right lower lobe; RM, right middle lobe; RU, right upper lobe; SCC, squamous cell carcinoma; SD, standard deviation; SUV, standardized uptake value.

**Table 2** shows the characteristics of the 2 groups after PSM. After PSM, the SMDs for almost all measured covariates were below the commonly accepted threshold (e.g., 0.1), indicating that the 2 groups were well balanced. This suggests that our matching procedure effectively minimized baseline differences between patients in the nondisposable and disposable knife groups, allowing for a more reliable comparison of outcomes.

**Table 2.** Clinical and Pathological Findings of All Patients After Propensity Score Matching

		Automatic stapling device		
Factors		Nondisposable (n = 78) (%)	Disposable (n = 78) (%)	SMD
Age	Median ± SD	70.5 ± 10.1	71.5 ± 8.7	0.061
Sex	Male	39 (50.0)	37 (47.4)	0.052
	Female	39 (50.0)	41 (52.6)	−0.052
Smoking	Never	43 (55.1)	42 (53.9)	0.026
	Ever	35 (44.9)	36 (46.1)	−0.026
Histological type	AD	66 (84.7)	67(85.9)	−0.037
	SCC	9 (11.5)	9 (11.5)	0.000
	Others	3 (3.8)	2 (2.6)	0.073
Clinical tumour size	Median ± SD (mm)	17.5 ± 7.4	17.0 ± 6.2	−0.070
Clinical invasive size	Median ± SD (mm)	7.5 ± 8.3	6.5 ± 8.4	−0.093
GGO	(+)	54 (69.2)	57 (73.1)	−0.085
	(−)	24 (30.8)	21 (26.9)	0.085
Pathological tumour size	Median ± SD (mm)	15.0 ± 6.9	15.0 ± 6.5	0.071
Vascular invasion	(+)	5 (6.4)	4 (5.1)	0.055
	(−)	73 (93.6)	74 (94.9)	−0.055
Lymphatic invasion	(+)	4 (5.1)	5 (6.4)	−0.055
	(−)	74 (94.9)	73 (93.6)	0.055
Histological grade	G1	49 (62.8)	50 (64.1)	−0.027
	G2	22 (28.2)	24 (30.8)	−0.057
	G3	6 (7.7)	3 (3.8)	0.166
	G4	1 (1.3)	1 (1.3)	0.000
Surgical procedure	Wedge	42 (53.8)	38 (48.7)	0.103
	Segmentectomy	36 (46.2)	40 (51.3)	−0.103
Number of staples used	Median ± SD	4.0 ± 1.2	3.0 ± 1.0	−0.290
Additional resection	(+)	1 (1.3)	1 (1.3)	0.000
	(−)	77 (98.7)	77(98.7)	0.000
Tumour location	RU	14 (17.9)	15 (19.2)	−0.065
	RM	2 (2.6)	2 (2.6)	0.000
	RL	22 (28.2)	22 (28.2)	0.000
	LU	23(29.5)	23 (29.5)	0.000
	LL	17 (21.8)	16 (20.5)	0.031
CEA value	Median ± SD	2.6 ± 3.5	2.5 ± 2.2	0.033
SUV max value	Median ± SD	1.4 ± 2.3	1.3 ± 2.7	0.006

Abbreviations: Median±standard deviation, Median[minimum-maximum]; SMD, standardized mean difference; AD, adenocarcinoma; CEA, carcinoembryonic antigen; GGO, ground-glass opacity; LL, left lower lobe; LU, left upper lobe; RL, right lower lobe; RM, right middle lobe; RU, right upper lobe; SCC, squamous cell carcinoma; SD, standard deviation; SUV, standardized uptake value.

Stapling cartridge lavage cytology was performed in 175 patients (65.1%), and rapid on-site cytological evaluations were performed in 108 patients (40.1%). Additional resection was performed in 3 patients, in whom a rapid on-site cytological evaluation was positive. Of the 4 patients in whom additional resection was performed, 3 showed intraoperative stapling cartridge lavage cytology (+), and the remaining 1 underwent additional resection due to insufficient margins during surgery. Twelve patients underwent stapling cartridge lavage cytology (+), of which 9 were found to be stapling cartridge lavage cytology (+) after surgery. Six of these 9 patients had local recurrence, 2 of whom underwent chemotherapy and 1 underwent additional lobectomy after recurrence. Two of the 3 patients without recurrence underwent postoperative radiation therapy targeting the stump.

**Figure 3.**
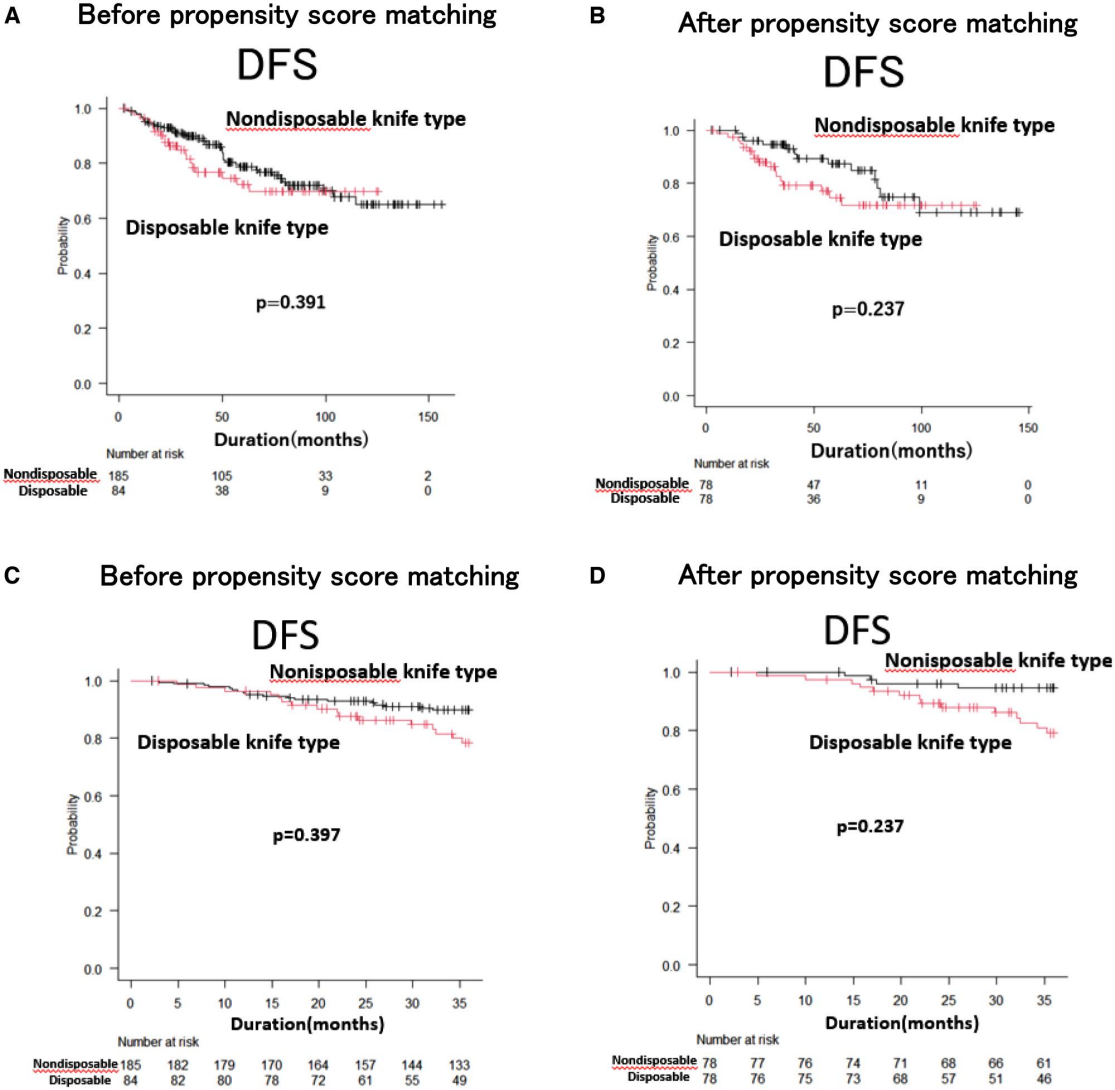
Disease-Free Survival (DFS) Curves for Lung Cancer Patients Who Underwent Pulmonary Resection According to the Type of Automatic Stapling Device Used (Nondisposable Knife Type vs Disposable Knife Type). (A and B) The DFS curves over the entire observation period, before and after propensity score matching (PSM), respectively. (C and D) DFS curves restricted to the first 3 years of follow-up, before and after PSM, respectively.

**Figure 4.**
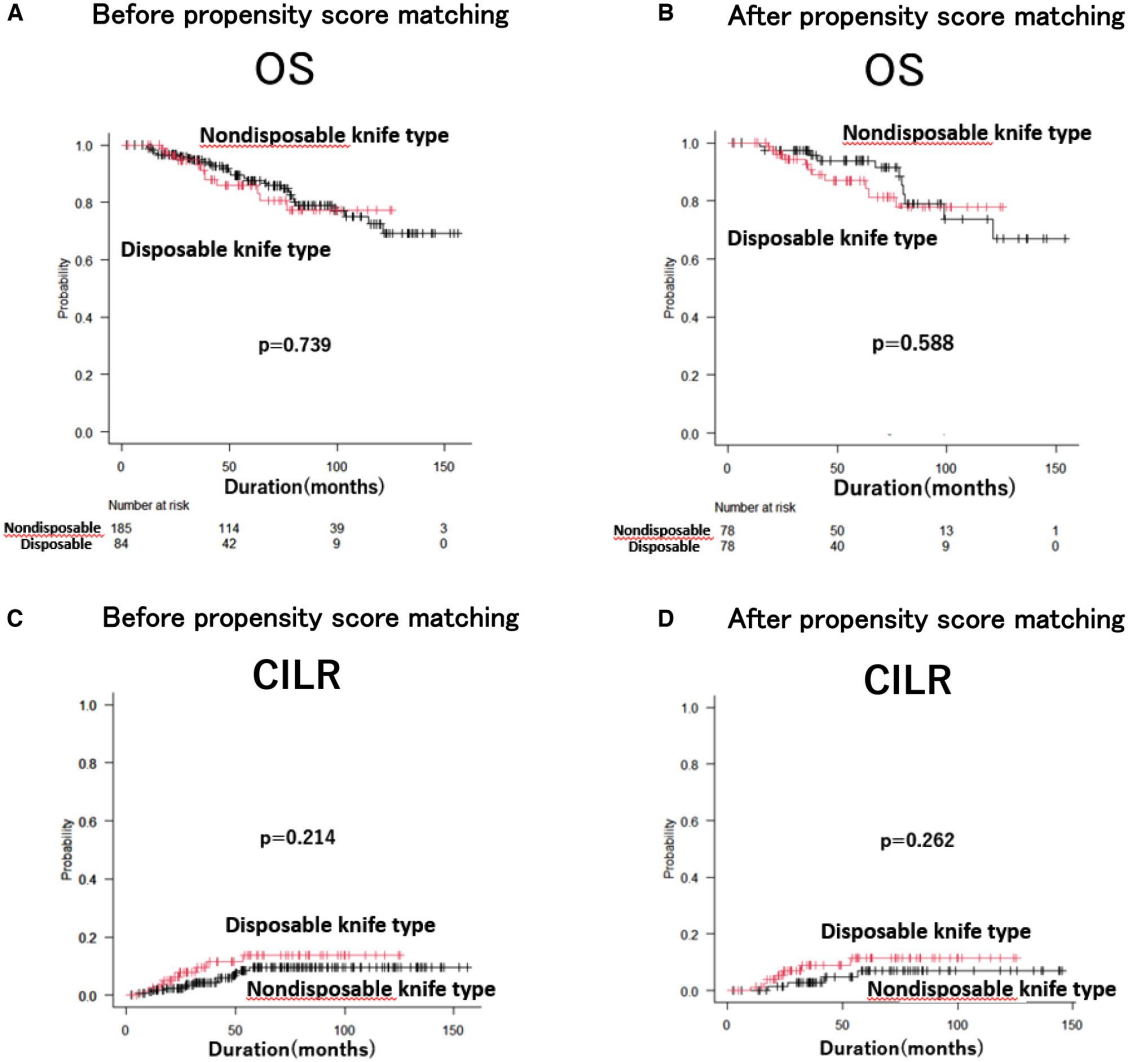
Overall Survival (OS) Curves for Lung Cancer Patients Who Underwent Pulmonary Resection According to the Type of Automatic Stapling Device Used (Nondisposable Knife Type vs Disposable Knife Type). (A and B) The OS curves over the entire observation period, before and after propensity score matching (PSM), respectively. Cumulative incidence of local recurrence (CILR) in the included patients, stratified by the type of automatic stapling device that was used (Nondisposable knife type vs Disposable knife type). (C) CILR in 22 patients before PSM. (D) CILR in 11 patients after PSM.

Local recurrence events were analysed using Gray’s competing-risk model and linearized risk analysis, considering death as a competing risk. Disease-free survival, which includes local recurrence events as part of the composite outcome, was analysed using Kaplan-Meier curves and log-rank tests. The median follow-up period in this study was 5.9 years (range: 0.2-13.0). There was no difference in DFS between the nondisposable and disposable knife types before (**Figure 3A**) and after (**Figure 3B**) PSM (before matching: *P* = .391, after matching: *P* = .237). Before PSM, the nondisposable knife type (*n* = 185) experienced 41 local recurrence events over 978.39 person-years, corresponding to a linearized risk of 4.19 events per 100 person-years. In contrast, the disposable knife type (*n* = 84) had 20 events over 367.27 person-years (5.44 events per 100 person-years), yielding a rate ratio of approximately 1.30 (95% CI, 0.76-2.22; *P* = .337). After PSM (78 patients per group), the nondisposable knife type showed 17 events over 346.26 person-years (4.91 events per 100 person-years), whereas the disposable knife type had 13 events over 418.59 person-years (3.11 events per 100 person-years), resulting in a rate ratio of about 1.58 (95% CI, 0.77-3.25; *P* = .213). In both analyses, these differences were not statistically significant. Although initial Kaplan-Meier curves suggested a slight divergence favouring nondisposable knife type in the early follow-up period, additional analyses restricted to events occurring within the first 3 years demonstrated no significant difference in DFS between nondisposable and disposable knife types, both before (*P* = .397) and after (*P* = .237) PSM (**Figure 3C** and **D**). There was no difference in overall survival between the 2 groups before (**Figure 4A**) and after (**Figure 4B**) PSM (*P* = .739, *P* = .588, respectively). **Figure 4C** shows that there was no difference in the cumulative incidence among the 22 patients with local recurrence among the 269 patients before PSM (*P* = .214). **Figure 4D** shows that there was no difference in the CILR among the 11 matched patients after PSM (*P* = .262).

Among 269 patients before PSM, 22 patients developed local recurrence (surgical margin recurrence, *n* = 20; pleural dissemination, *n* = 2). Among the 20 patients of surgical margin recurrence, 13 patients were treated using a nondisposable knife type automatic stapling device and 7 patients were treated using a disposable knife type automatic stapling device.

## DISCUSSION

In this study, using PSM to standardize background factors, we investigated the effect of the type of automatic stapling device on local recurrence on radical sublobar resection. As a result, it became clear, for the first time, that the incidence of local recurrence did not differ according to the type of automatic stapling device used in thoracic surgery.

In the field of gastrointestinal surgery, Ohki et al found that isolated cancer cells attached to 6.9% of staples used in gastric cancer surgery and reported that the types of recurrence included recurrence at the resection line, peritoneal dissemination, and port site recurrence.^2^ However, there are currently 2 types of automatic stapling devices in use, and no reports have focused on the differences between them.

A post hoc power analysis was conducted to evaluate whether the sample size was sufficient to detect a meaningful difference between groups. Using a moderate effect size (Cohen’s d = 0.5), a significance level of 0.05, and a statistical power of 0.8, we determined that a minimum of 64 patients per group would be required to detect a statistically significant difference. Given that our study included 78 patients per group after PSM, the sample size is considered sufficient to detect clinically meaningful differences.

The 2 groups were generally well balanced in background characteristics, with only small to moderate imbalances observed in certain variables before PSM. Nondisposable knife devices were slightly more likely to be used for patients with larger pathological tumour diameters (SMD = 0.159) and those undergoing wedge resection (SMD = 0.237). The proportion of patients who required additional intraoperative resection showed little difference between the 2 groups, both before (SMD = 0.036) and after (SMD = 0) matching. However, the nondisposable group tended to have a higher number of staples used (SMD = 0.280 before PSM, 0.29 after PSM), possibly reflecting that the nondisposable device could not be length-adjusted for each use. These comparisons are detailed in **Tables 1** and **2**. Regarding CILR, even in the 11 patients who experienced local recurrence among the 156 patients after PSM, no difference was observed between the 2 groups (*P* = .262).

The median follow-up period in this study was 5.9 years (range: 0.2-13.0), and the overall local recurrence rate was 8.2%. In the JCOG0802/WJCOG4607L study (median follow-up period: 7.3 years [range 0.0 to 10.9]), in patients with a tumour diameter of ≤2 cm and a consolidation-to-tumour ratio of >0.5, the local recurrence rate after segmentectomy was reported to be 10.5%.^17^ The local recurrence rate in this study was comparable to rates reported in previous studies.

The usefulness of stapling cartridge lavage cytology in sublobar resection has been reported.^19^ Of the 9 patients with positive postoperative staple cartridge washing cytology, 2 received prophylactic radiation therapy targeting the surgical margins, and 6 patients experienced local recurrence without additional postoperative therapy. Based on these results, we believe that stapling cartridge lavage cytology should be performed during sublobar resection. Additionally, although washing the stapler cartridge with distilled water helps clear any residual tissue or blood, it may also exert a cytotoxic effect on tumour cells, which could have influenced the outcomes observed in our study.^20^

The present study was associated with several limitations. Out of the 22 patients who experienced local recurrence, only 6 had their margins measured in their pathology examination. Although margin measurements were not available for all cases, intraoperative evaluations ensured that an adequate margin was achieved in every procedure. The surgical team carefully confirmed that sufficient margins were maintained during resection to minimize the risk of local recurrence. It has been reported that there is a significant correlation between the distance of the surgical margin and recurrence; therefore, the margins should be accurately evaluated.^21^ Regarding the indications for sublobar resection, this study focused on lesions with a clinical invasive size of ≤3 cm. Segmentectomy was proven to be noninferior to lobectomy for solid-predominant NSCLC measuring ≤2 cm in diameter^17^; however, there is no scientific evidence for sublobar resection for solid diameters of 2-3 cm. A non-inferiority trial of segmentectomy versus lobectomy for stage IA3 pure solid tumours^22^ is currently ongoing, and the results are likely to provide clues to solve this problem. Furthermore, this was a retrospective observational study conducted at a single facility with a relatively small number of patients. However, we acknowledge future larger-scale prospective studies may further validate our findings.

## CONCLUSION

In this study, the recurrence rate did not differ according to the type of automatic stapling device that was used. Further verification in a larger randomized controlled trial is required.

## Data Availability

All data have been included in this article. Further inquiries can be directed to the corresponding author.

## References

[1] Hashimoto K , Takahashi T, Suzuki C. Micrometastasis in resected lungs of lung cancer patients. Gan. 1976;67:717-723. 1017586

[2] Ohki A , Takagi T, Kojima Y, et al Intragastric free cancer cells may be attached to automatic staplers during anastomosis in patients with gastric cancer. World J Surg Oncol. 2024;22:9. 38172834 10.1186/s12957-023-03285-2 PMC10765920

[3] Maurizi G , D'Andrilli A, Ciccone AM, et al Margin distance does not influence recurrence and survival after wedge resection for lung cancer. Ann Thorac Surg. 2015;100:918-925. 26209486 10.1016/j.athoracsur.2015.04.064

[4] Sawabata N. Tumor size, margin distance rate, and margin cytologic results influence recurrence and survival after wedge resection for lung cancer. Ann Thorac Surg. 2016;101:1241-1242. 26897223 10.1016/j.athoracsur.2015.08.020

[5] Mohiuddin K , Haneuse S, Sofer T, et al Relationship between margin distance and local recurrence among patients undergoing wedge resection for small (≤2 cm) non-small cell lung cancer. J Thorac Cardiovasc Surg. 2014;147:1169-1175. 24507406 10.1016/j.jtcvs.2013.11.056

[6] Sawabata N , Ohta M, Matsumura A, et al; Thoracic Surgery Study Group of Osaka University. Optimal distance of malignant negative margin in excision of nonsmall cell lung cancer: a multicenter prospective study. Ann Thorac Surg. 2004;77:415-420. 14759408 10.1016/S0003-4975(03)01511-X

[7] Sawabata N , Matsumura A, Ohota M, et al; Thoracic Surgery Study Group of Osaka University. Cytologically malignant margins of wedge resected stage I non-small cell lung cancer. Ann Thorac Surg. 2002;74:1953-1957. 12643379 10.1016/s0003-4975(02)03993-0

[8] Sawabata N , Mori T, Iuchi K, Maeda H, Ohta M, Kuwahara O. Cytologic examination of surgical margin of excised malignant pulmonary tumor: methods and early results. J Thorac Cardiovasc Surg. 1999;117:618-619. 10047671 10.1016/s0022-5223(99)70347-8

[9] Miyoshi T , Yoshida J, Aokage K, Tane K, Ishii G, Tsuboi M. Stapling cartridge lavage cytology in limited resection for pulmonary malignant tumors: assessment of cytological status of the surgical margin. Heliyon. 2019;5:e01240. 30815608 10.1016/j.heliyon.2019.e01240 PMC6378348

[10] Higashiyama M , Kodama K, Takami K, et al Intraoperative lavage cytologic analysis of surgical margins as a predictor of local recurrence in pulmonary metastasectomy. Arch Surg. 2002;137:469-474. 11926957 10.1001/archsurg.137.4.469

[11] Miyoshi K , Fuchimoto S, Ohsaki T, et al Suture line recurrence in jejunal pouch replaced after total gastrectomy for gastric cancer. Gastric Cancer. 1999;2:194-197. 11957096 10.1007/s101200050046

[12] Nishimura M , Honda I, Watanabe S, Nagata M, Souda H, Miyazaki M. Recurrence in jejunal pouch after proximal gastrectomy for early upper gastric cancer. Gastric Cancer. 2003;6:197-201. 14520535 10.1007/s10120-003-0242-7

[13] Namikawa T , Kobayashi M, Okamoto K, et al Recurrence of gastric cancer in the jejunal pouch after completion gastrectomy. Gastric Cancer. 2007;10:256-259. 18095082 10.1007/s10120-007-0441-8

[14] Shinohara T , Kashiwagi H, Nakada K, et al Suture line recurrence in the jejunal pouch after curative proximal gastrectomy for gastric cancer: report of two cases. Hepatogastroenterology. 2007;54:1902-1904. 18019745

[15] Polychronidis A , Laftsidis P, Giatromanolaki A, Perente S, Bounovas A, Simopoulos C. Suture-line recurrence at a jejunojejunal anastomosis after gastrectomy for gastric cancer. Gastric Cancer. 2008;11:59-63. 18373179 10.1007/s10120-007-0446-3

[16] Dolan DP , Lee DN, Kucukak S, et al Salvage surgery for local recurrence after sublobar surgery in stages I and II non-small cell lung cancer. J Surg Oncol. 2022;126:814-822. 35603966 10.1002/jso.26925

[17] Saji H , Okada M, Tsuboi M, et al; West Japan Oncology Group and Japan Clinical Oncology Group. Segmentectomy versus lobectomy in small-sized peripheral non-small-cell lung cancer (JCOG0802/WJOG4607L): a multicentre, open-label, phase 3, randomised, controlled, non-inferiority trial. Lancet. 2022;399:1607-1617. 35461558 10.1016/S0140-6736(21)02333-3

[18] Bierley JD , Gospodarowicz MK, Wittekind C. TNM Classification of Malignant Tumours. 8th ed. Hoboken, NJ: Wiley; 2017.

[19] Utsumi T , Sawabata N, Inoue M, Okumurae M. Optimal sampling methods for margin cytology examination following lung excision. Interact CardioVasc Thorac Surg. 2010;10:434-436. 19955172 10.1510/icvts.2009.212142

[20] Huguet EL , Keeling NJ. Distilled water peritoneal lavage after colorectal cancer surgery. Dis Colon Rectum. 2004;47:2114-2119. 15657663 10.1007/s10350-004-0788-4

[21] Kamimura G , Ueda K, Suzuki S, Maeda K, Hakamada H, Sato M. Intraoperative computed tomography of a resected lung inflated with air to verify safety surgical margin. Quant Imaging Med Surg. 2022;12:1281-1289. 35111623 10.21037/qims-21-562 PMC8739102

[22] Kamigaichi A , Hamada A, Tsuboi M, et al A multi-institutional, randomized, phase iii trial comparing anatomical segmentectomy and lobectomy for clinical stage IA3 pure-solid non-small-cell lung cancer: West Japan Oncology Group Study WJOG16923L (STEP UP Trial). Clin Lung Cancer. 2024;25:384-388.e1. 38360496 10.1016/j.cllc.2024.01.004

